# Identification, expression, and functional analysis of *CLE* genes in radish (*Raphanus sativus* L.) storage root

**DOI:** 10.1186/s12870-015-0687-y

**Published:** 2016-01-27

**Authors:** Maria S. Gancheva, Irina E. Dodueva, Maria A. Lebedeva, Varvara E. Tvorogova, Alexandr A. Tkachenko, Ludmila A. Lutova

**Affiliations:** Department of Genetics and Biotechnology, Saint-Petersburg State University, Saint-Petersburg, 199034 Russia

**Keywords:** *Raphanus sativus*, *Raphanus raphanistrum*, CLE peptides, storage root, Cambium, Xylem

## Abstract

**Background:**

Radish (*Raphanus sativus* L.) is a widespread agricultural plant forming storage root due to extensive secondary growth which involves cambium proliferation and differentiation of secondary conductive tissues. Closely related to the model object *Arabidopsis thaliana*, radish is a suitable model for studying processes of secondary growth and storage root development. CLE peptides are a group of peptide phytohormones which play important role in the regulation of primary meristems such as SAM, RAM, and procambium, as well as secondary meristems. However, the role of CLE peptides in lateral growth of root during storage root formation has not been studied to date.

**Results:**

In present work we studied the role of CLE peptides in the development of storage root in radish. We have identified 18 *CLE* genes of radish (*RsCLEs*) and measured their expression in various plant organs and also at different stages of root development in *R. sativus* and *Raphanus raphanistrum*—its close relative which does not form storage root. We observed significant decline of expression levels for genes *RsCLE1*, *2*, *11*, *13*, and *16*, and also multifold increase of expression levels for genes *RsCLE19*, and *41* during secondary root growth in *R. sativus* but not in *R. raphanistrum*. Expression of *RsCLE 2*, *19*, and *41* in *R. sativus* root was confined to certain types of tissues while *RsCLE1*, *11*, *13*, and *16* expressed throughout the root. Experiments on overexpression of *RsCLE2*, *19* and *41* or treatment of radish plants with synthetic CLE peptides revealed that CLE19 and CLE2 increase the number of xylem elements, and CLE41 induces the formation of extra cambium foci in secondary xylem. Expression levels of *RsCLE2* and *19* strongly decrease in response to exogenous cytokinin, while auxin causes dramatic increase of *RsCLE19* expression level and decrease of *RsCLE41* expression.

**Conclusions:**

Our data allow us to hypothesize about the role of *RsCLE2*, *19* and *41* genes in the development of storage root of *Raphanus sativus*, e.g. *RsCLE19* may play a role in auxin-dependent processes of xylem differentiation and *RsCLE41* stimulates cambium activity.

**Electronic supplementary material:**

The online version of this article (doi:10.1186/s12870-015-0687-y) contains supplementary material, which is available to authorized users.

## Background

The family of CLE (CLAVATA3/ENDOSPERM SURROUNDING REGION) peptide phytohormones includes small (less than 15 kD) mobile peptides. Being translated as precursors of about 100 amino acid (aa) residues, mature CLE peptides include the only conserved C-terminal CLE domain of 12–14 aa [1]. CLE peptides bind to CLV1-like receptor protein kinases and trigger the poorly studied pathway of signal transduction, which eventually changes the expression level of *WOX* genes, central regulators of stem cell pools in different meristems [2]. CLE peptides were found in various plant species [3] and also outside of the plant kingdom, in some parasitic nematodes [4]. *Arabidopsis thaliana* genome contains 32 *CLE* genes with different spatial and temporal expression patterns [5, 6]. It is accepted that functions of most CLE peptides in the meristems consist in the negative regulation of stem cell proliferation and thereby maintaining meristem size. An exception from this is a small group of TDIF (TRACHEARY ELEMENT DIFFERENTIATION INHIBITORY FACTOR)-like CLE peptides, which do not suppress cell proliferation in the meristems or even stimulate it in the procambium and cambium [7]. According to [8], CLE peptides which do not suppress meristematic cell proliferation, are combined in the group B, whereas other CLE peptides form group A. B-type CLEs differ from other CLE peptides by the specific amino acid composition of the CLE domain and some features in post-translational modification [7]. Among 32 CLE peptides of *Arabidopsis*, closely related CLE41, CLE44, and CLE42 belong to the group B [7, 8]. Some *Arabidopsis* CLE peptides function as central regulators of apical and lateral primary meristems: in particular, CLV3 peptide is required for correct SAM development [9]; CLE40 has similar function in RAM [10]; CLE41/CLE44 controls the development of procambium and cambium [11]. In addition, several CLE peptides regulate other types of meristems, e.g. A-type CLEs MtCLE13 and PsCLE13 regulate development of nodule meristems in *Medicago truncatula* and *Pisum sativum* respectively [12, 13]. Moreover, CLE peptides are supposed to regulate other developmental processes, like early embryogenesis [14] or vessel development [11, 15]. Taking all of this into account, we can hypothesize that CLEs may function as regulators of storage root development in radish.

Radish (*Raphanus sativus* L., 2*n* = 2*x* = 18) is an important vegetable crop because of its edible storage root. Several variations of cultivated radish are known, e.g. cherry radish (*R. sativus* L. var. *radicola* Pers), oil radish (*R. sativus* L. var. *oleifera*), feed radish (*R. sativus* L. var. *caudatus*), black radish (*R. sativus* L. var. *niger*), and large root radish, or daikon (*R. sativus* L. var. *longipinnatus* Bailey). According to most opinions, *R. sativus* L. was originated from wild radish *R. raphanistrum* L. or was derived by hybridization between *R. maritimus* and *R. landra* [16, 17]. In recent years, the RadishBase [18], a genomic database of radish, was developed [19], and comprehensive analysis of expressed sequence tags from cultivated and wild radish was performed [20]. A genetic collection of *R. sativus* L. var. *radicola* Pers is maintained in Saint-Petersburg State University (Russia) since 1970. This collection was derived from single plants belonging to three radish cultivars (Saxa, Virovskyi Belyi, Ledianaya Sosulka) by inbreeding during more than 40 generations. At present, the collection includes 33 self-compatible highly inbred lines; some of them demonstrate different morphological abnormalities (e.g. dwarfism, agravitropic growth, tumor formation etc.) [21, 22].

**Fig. 1:**
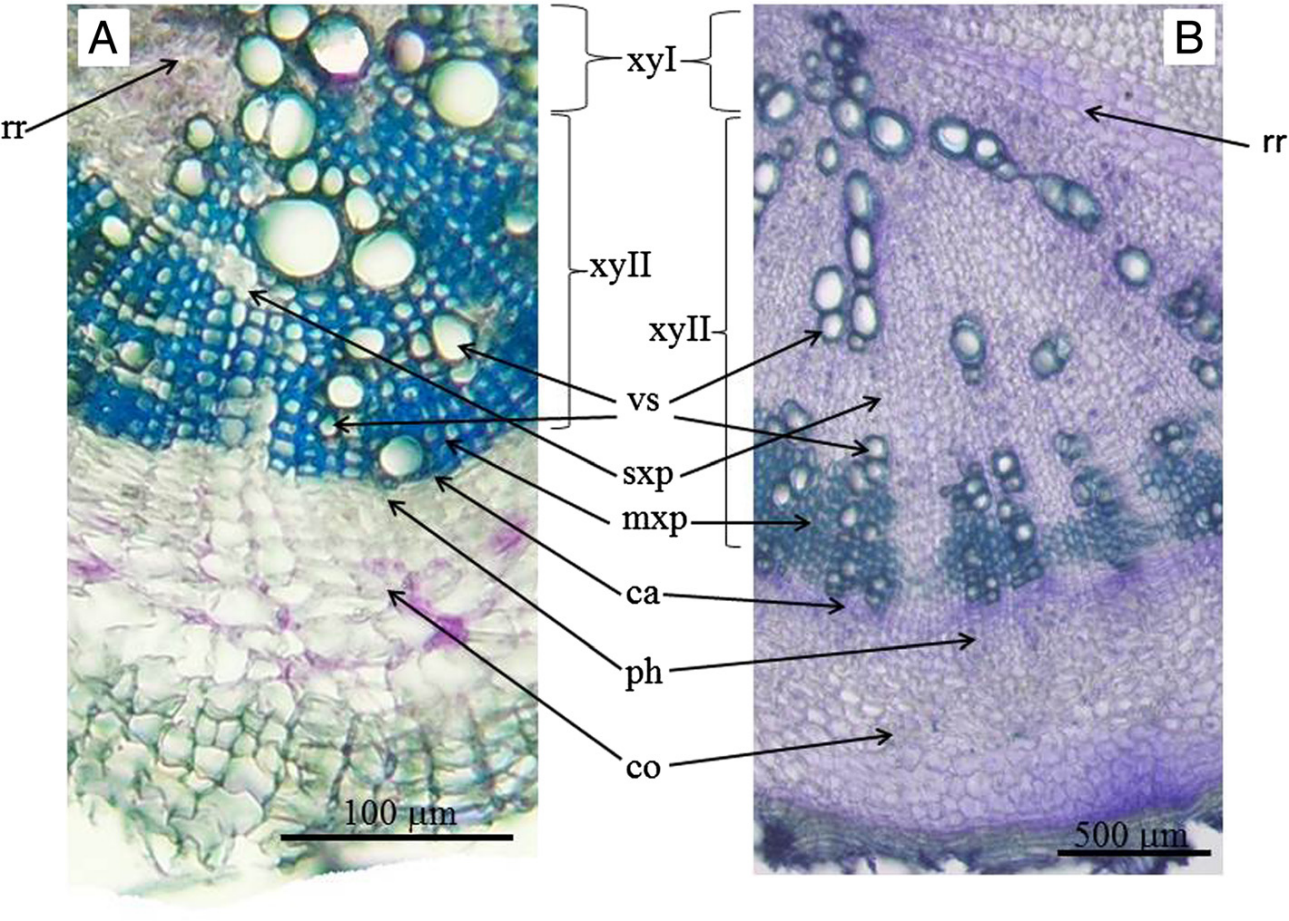
Comparison of the anatomical structure of *Raphanus raphanistrum* (**a**) and *Raphanus sativus* (**b**) roots (30 day old plants). xyI—primary xylem, xyII—secondary xylem, ca—cambium, ph—phloem, co—cortex, vs—vessels, mxp—mechanical xylem parenchyma, sxp—storage xylem parenchyma, rr—radial ray

Unlike its close relative *Arabidopsis thaliana*, *R. sativus* has a peculiar developmental feature, namely the formation of a storage root (so called crop-root). Therefore, radish is a perspective model to study mechanisms of storage root development. It is known that radish crop-root originates from the hypocotyl and upper part of root [23] due to the secondary thickening as a result of cambium activity [24, 25]. The secondary thickening involves proliferation of vascular cambium and differentiation of secondary xylem and phloem inwards and outwards from cambium. In case of radish the proportion of phloem and xylem differentiation is shifted towards xylem (so called xylem-type storage root). As a result, mature radish storage root is composed of a large zone of secondary xylem tissue surrounded by a narrow cambial zone, band of secondary phloem, and outer layer of secondary cortex derived from pericycle [25]. Zone of secondary xylem in radish storage root is quite clearly separated into central and periphery parts. Periphery part of secondary xylem zone includes vessels surrounded by rows of small-cell thick-walled parenchyma cells which perform predominantly mechanical function (mechanical parenchyma); vessels and mechanical parenchyma form bands, separated by wide radial rays of thin-walled parenchyma cells filled with starch grains [26]. Central part of secondary xylem in the root of *R. sativus* includes more rare vessels lying in the mass of the thin-walled storage parenchyma (Fig. 1b). Some researchers [26, 27] noted that zone of secondary xylem in radish storage root (mainly an inner region of secondary xylem) also includes numerous little foci of cambium-like secondary meristem (so called “meristematic foci”) which are maintained for a limited time and give rise to a small number of tertiary conductive elements. On the other hand, *R. raphanistrum*, presumable ancestor of *R. sativus* [16, 17] demonstrate less extensive secondary thickening and does not form storage root. The main differences in the anatomical structure of *R. sativus* and *R. raphanistrum* are less extensive zone of secondary xylem and significantly fewer cells of the storage parenchyma in the root of *R. raphanistrum* (Fig. 1).

The main goal of our investigation was determining of the role of CLE peptides in the development of storage root in radish. In the present work we identified *Raphanus sativus RsCLE* genes based on their homology with corresponding *Arabidopsis thaliana AtCLE* genes. Then, we analyzed the expression of *RsCLE* genes in different parts of seedling, and also in the roots at different stages of development in *R. sativus* which forms storage root and in *R. raphanistrum* which does not. In addition, we studied the effect of overexpression of certain *RsCLE* genes of A- and B-types as well as the effect of exogenously applied CLE peptides on radish storage root development. Our data suggest the involvement of *RsCLE2*, *19*, and *41* genes in the development of radish storage root. Finally, we studied the effect of exogenous cytokinin and auxin on the expression of several A- and B-type *RsCLE* genes. The results obtained indicate that CLE-peptides, cytokinins and auxins may interact during the development of radish storage root.

## Results and discussion

### Identification of *Raphanus sativus CLE* (*RsCLE*) genes

The genome of *Arabidopsis thaliana* contains 32 *CLE* genes [1], and each of them has its own unique expression pattern [5, 6]. We identified radish homologues of the most part of *Arabidopsis AtCLE* genes whose expression, according to [6], was observed in the root (except for *AtCLE6*, *7*, and *44*). In total we have identified 16 *RsCLE* genes homologous to *Arabidopsis* A-type *CLE* genes (*RsCLE1*, *2*, *4*, *5*, *11*, *12*, *13*, *16*, *17*, *19*, *20*, *22*, *25*, *26*, *27*, and 40) and 2 *RsCLE*s that are homologous to *Arabidopsis* B-type *CLE* genes (*RsCLE41*, and *RsCLE42*). CDS (coding sequences) of *RsCLEs* identified demonstrated 72.9–90.7 % of identity with corresponding *AtCLE* genes (Table 1) and also with *CLE*-like sequences of *Brassica rapa* [28]. However, we failed to find radish homologues of *AtCLE6*, *AtCLE7*, and *AtCLE44* genes: all primer sets that were designed based on corresponding genes of *Arabidopsis* did not give any PCR product on radish DNA or anneal to other closely related *RsCLE*s. Therefore, we suppose that there are no homologues of *CLE6*, *CLE7*, and *CLE44* genes in *Raphanus sativus* genome. The sequences homologous to *AtCLE6*, *AtCLE7*, and *AtCLE44* genes are also absent among identified *Brassica rapa* sequences [28].

**Table 1:** Identified *Raphanus sativus CLE* (*RsCLE*) genes

Gene	Length of CDS, bp	Identity with CDS of *Arabidopsis* genes, %	CLE-domain sequence^a^	GenBank accession number
*RsCLE1*	234	80,3	RLSPGGPDPRHH	KF965525
*RsCLE2*	240	84,6	RLSPGGPDPQHH	KF965526
*RsCLE4*	249	87,6	RLSPGGPDPRHH	KF965533
*RsCLE5*	246	80,1	RVSPGGPDPQHH	KF965527
*RsCLE11*	297	84,0	R**L**VPSGP**R**PLHH	KF965528
*RsCLE12*	366	79,0	RRVPSGPNPLHH	KT803936
*RsCLE13*	315	74,5	RLVPSGPNPLHH	KF965529
*RsCLE16*	306	75,6	RLVHTGPNPLHN	KF965534
*RsCLE17*	280	75,2	RVV**R**TGPNPLHN	KF965535
*RsCLE19*	228	86,4	R**I**IPTGPNPLHN	KF965530
*RsCLE20*	255	85,1	RKVKTGSNPLHN	KF965536
*RsCLE22*	306	82,4	RRVFTGPNP**S**H**S**	KF965537
*RsCLE25*	246	90,7	RKVPNGPDPIHN	KF965538
*RsCLE26*	346	74,6	RKVPRG**S**DPIHN	KT803934
*RsCLE27*	282	72,9	R**P**VPS**G**PDPLHN	KT803935
*RsCLE40*	261	84,7	RQVPTGSDPLHH	KF965539
*RsCLE41*	300	86,7	HEVPSGPNPISN	KF965531
*RsCLE42*	255	84,3	HGVPSGPNPISN	KF965532

^a^amino acid residues which differ from the amino acids in the CLE domain of *Arabidopsis* are marked by bold underscored text

Predicted sequences of CLE domains of most of *RsCLEs* are similar to those of *AtCLEs*. Exceptions are *RsCLE11*, *17*, *19*, *22*, *26*, and *27* whose CLE domains presumably differ from *Arabidopsis* in one or two amino acids (Table 1). Like *AtCLE* genes, *RsCLEs* gene sequences are short, their coding sequence lengths are 240–330 bp. Most of *RsCLEs*, like *AtCLEs*, lack introns, with the exception of *RsCLE40*, which has two introns like its homologue *AtCLE40* [10].

According to previous data, substitution of the glycine residue in the sixth position of CLE domain has the most pronounced effect on the function of CLE peptides in *Arabidopsis* [29]. The glycine at sixth position is highly conserved in CLE-domains of all *Arabidopsis* CLE peptides; the only exception is AtCLE27 that has cysteine at sixth position of CLE-domain. However, all identified radish CLEs contain conserved glycine residue in the sixth position of CLE-domain.

### qRT-PCR analysis of *RsCLE* genes expression

Using qRT-PCR method, we analyzed *RsCLE* genes expression in different organs of *R. sativus* and *R. raphanistrum* seedlings. Most of *RsCLEs* expressed in all parts of plants, but some of them were expressed in a certain plant organ only, e.g. *RsCLE1*, *RsCLE2*, and *RsCLE13* demonstrated root-specific expression (Additional file 1: Table S1 and Additional file 2: Figure S1). According to literature data [5, 6] corresponding *AtCLE* genes have similar expression pattern in seedlings. Among studied *RsCLEs*, expression of *RsCLE16* was the strongest in root and hypocotyl of *R. sativus* seedlings, whereas *RsCLE4* and *RsCLE17* were the most weakly expressed genes (Additional file 2: Figure S1). Expression patterns of some *RsCLEs* slightly differed in *R. sativus* and *R. raphanistrum* seedlings—e.g. *RsCLE19* expressed at high level in the root of *R. sativus* but not in the root of *R. raphanistrum*.

Then we analyzed the expression of *RsCLEs* in the root and hypocotyl in two different lines of *R. sativus* and in *R. raphanistrum* at different developmental stages: 7-day old seedling, 15- and 30-day old plants (stages 1, 2, 3, correspondingly). In *R. sativus* 15-day old plants at four leaves stage, storage root formation is started through the extensive root thickening, in 30-day old plants at rosette stage root thickening reaches the maximum [25]. In contrast to *R. sativus*, *R. raphanistrum* does not form a storage root, and therefore demonstrates less pronounced root thickening at the same developmental stages which gave us the reason to check whether *R. sativus* and *R. raphanistrum* differ in expression levels of *RsCLEs*

**Fig. 2:**
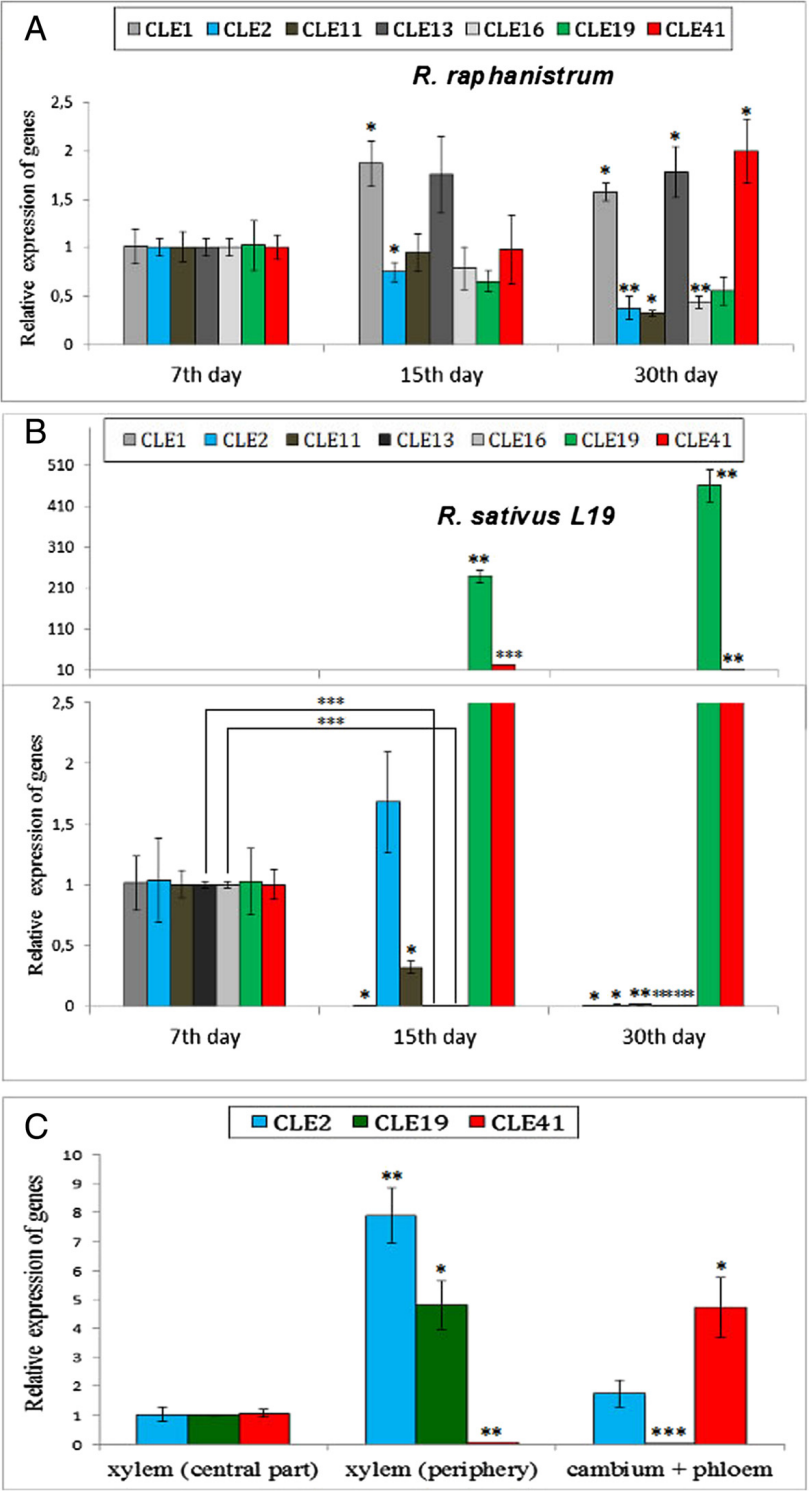
qRT-PCR analysis of *RsCLE* genes expression. **a**, **b** *RsCLE* genes expression in *Raphanus raphanistrum* (**a**) and *Raphanus sativus* (**b**) roots (line 27) at different stage of development. Expression levels are shown relative to the expression found in the root of 7-day old seedlings. **c** *RsCLE* genes expression in different tissues of *Raphanus sativus* storage root. Error bars indicate standard deviation of three technical repeats. (*P* < 0.05 - *, *P* < 0.01 - **, *P* < 0.001 - ***)

We revealed that expression levels of some *RsCLEs* were significantly increased or decreased in the roots and hypocotyls of 15- and 30-day old plants of both analyzed *R. sativus* lines, but not in the roots and hypocotyls of *R. raphanistrum* (Fig. 2a, b, and Additional file 3: Figure S2). Genes, whose expression increases tenfold and more in thickening storage root of *R. sativus*, included one A-type CLE gene (*RsCLE19*) (Welch’s *t*-test *p*-value < 0.001; *n* = 3), and one B-type CLE gene—(*RsCLE41*) (Welch’s *t*-test *p*-value < 0.001; *n* = 3). At the same time, expression levels of other five A-type *CLE* genes—*RsCLE1*, *2*, *11*, *13*, and *16* decreased significantly (Welch’s *t*-test *p*-values 0.0153, 0.0359, 0.0045, <0.0001, <0.0001, respectively; *n* = 3) during root thickening in *R. sativus* plants (Fig. 2a, b, and Additional file 3: Figure S2).

For more detailed expression analysis of *RsCLE1*, *2*, *11*, *13*, *16*, *19*, and *41* we dissected several zones in *R. sativus* storage root (central (1) and periphery (2) parts of secondary xylem, and also phloem + cambium (3)) and then analyzed *RsCLEs* expression in these zones. We found that *RsCLE1*, *11*, *13*, and *16* are expressed in all tissues across radish root, while *RsCLE2*, *19*, and *41* have zone-specific expression pattern. *RsCLE2* and *19* are expressed specifically in secondary xylem, and *RsCLE41* is expressed in the phloem and cambium zones. It is interesting that the expression of all studied *RsCLEs* was revealed in secondary meristem foci which are initiated in central part of xylem in radish storage root (Fig. 2c).

According to our results, three *RsCLEs* (*RsCLE2*, *19*, and *41*) specifically express in certain tissues that provide secondary growth of root (*RsCLE2* and *19* were expressed in secondary xylem, *RsCLE41* was expressed in phloem and cambium). Moreover, dramatic change of their expression levels was associated with the beginning of extensive root thickening in *R. sativus*. So, we suppose that these genes may participate in the regulation of storage root development.

In *Arabidopsis*, 23 of 29 *AtCLE* genes encoding A-type CLE peptides are also expressed in roots, and some of them demonstrate tissue-specific expression pattern [5, 6]. All *Arabidopsis* genes encoding B-type CLE peptides (e.g. *AtCLE41*) are expressed in the vascular tissues of all plant organs including the root [30, 31]; *AtCLE19* and *BnCLE19* genes of *Arabidopsis* and *Brassica* roots are expressed specifically in pericycle cells facing the protoxylem poles [32], and *AtCLE2*—in the primordial of lateral roots [6].

### Effect of altered expression levels of *RsCLEs* and of treatment with exogenous CLE peptides on radish storage root development

Many investigators have studied the changes in different aspects of plant development caused by altered expression levels of *CLE* genes, or by treatment of plants with exogenous CLE peptides. Most of such studies are focused mainly on SAM and RAM activity [6, 7, 33–35], some of them also consider cambium activity and vascular system development [7, 8, 11]. These experiments revealed that B-type CLE peptides (CLE41, 42, and 44 in *Arabidopsis*) stimulate proliferation of cambium cells via activation of *WOX4* gene expression, and also inhibit their differentiation into xylem. On the other hand, it is obvious that some A-type CLE peptides also take part in the development of vascular system; e.g. overexpression of *CLE19* gene in *Arabidopsis* and *Brassica* stimulated the differentiation of xylem, leading to the formation of xylem “islands” in the flower organs [32]; AtCLE10 inhibits vessel formation in *Arabidopsis* roots via repression of the expression of two type-A *Arabidopsis Response Regulators* (*ARR*s), *ARR5* and *ARR6*, whose products act as negative regulators of cytokinin signaling [15].

Thus, A- and B-type CLE peptides may control the balance between cambium cells proliferation and differentiation of xylem. Nevertheless, simultaneous overexpression of A- and B-type *CLE* genes, or plant treatment by CLE peptides from both groups leads to more pronounced stimulating effect on cambium cell proliferation than overexpression of only B-type *CLE* genes [8]. Therefore, interaction of A- and B-type CLEs in the control of vascular system development and root secondary growth is more complex than simple antagonism.

**Fig. 3:**
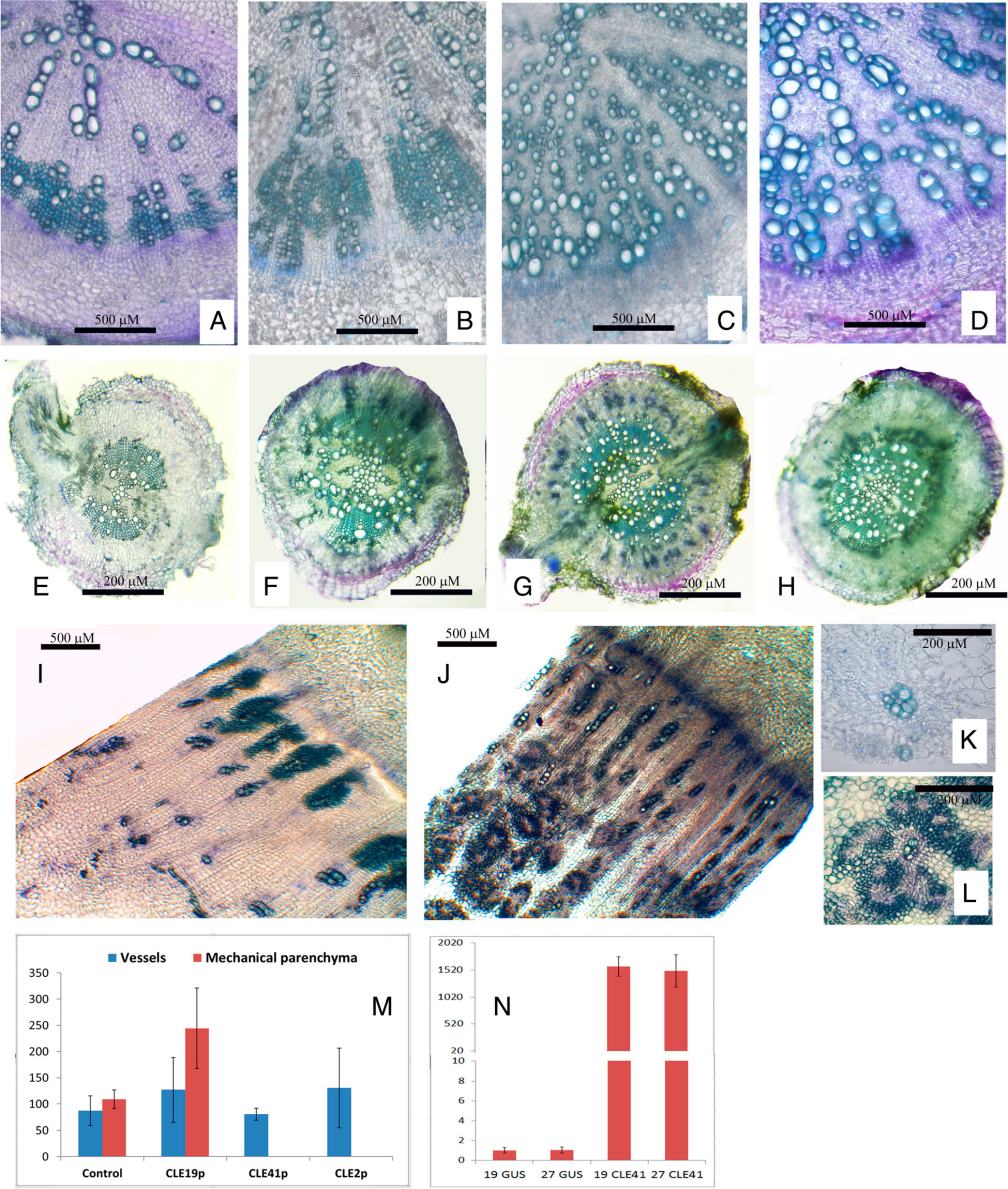
Effect of CLE-peptides on the development of *Raphanus raphanistrum* and *Raphanus sativus* roots. **a**-**h**—Effect of exogenous CLE peptides supplying. Transverse sections of *Raphanus sativus* line 19 (**a**-**d**) and *Raphanus raphanistrum* (**e**-**h**) roots of 15-day old plants after 7 days cultivation of medium with synthetic CLE-peptides: control (CLE41def) (**a**, **e**), CLE19p (**b**, **f**), CLE41p (**c**, **g**), and CLE2p (**d**, **h**). **i**-**m** Effect of *RsCLE41* overexpression on development of meristematic foci in the central part of xylem zone. **i**, **j** transverse sections of mature (30 day old) roots of *Raphanus sativus* line 19: **i**—*GUS* overexpression (control); **j** *RsCLE41* overexpression. **k**, **l**—meristematic foci in the central part of xylem zone of roots with *GUS* overexpression (**k**) and *RsCLE41* overexpression (**l**). **m** number of xylem elements on transverse section of *Raphanus sativus* root of 250 μM in diameter (*P* < 0.01 - **, *P* < 0.001 - ***). **n** qRT-PCR analysis of *RsCLE41* expression in *GUS*- overexpressing and *RsCLE41*-overexpressing *Raphanus sativus* roots (lines 19 and 27)

We have studied the effect of overexpression of several *RsCLEs*, encoding A-type (*RsCLE2* and *19*) and B-type (*RsCLE41*) CLE peptides on secondary root structure in radish. We also treated *R. sativus* and *R. raphanistrum* plants by synthetic CLE peptides CLE2, CLE19, and CLE41. Both experiments provided very similar results: plants with shifted quantity of a certain CLE peptide in the root (resulted from *CLE* overexpression or treatment by exogenous CLE) had altered development of storage root tissues (Fig. 3).

First, roots of *R. sativus* 15- and 30-day old plants with overexpression of *RsCLEs* or after treatment by CLE peptides demonstrated altered quantity of certain type of secondary xylem elements—mechanical xylem parenchyma, which is normally adjacent to vessels in mature storage root of radish. Unexpectedly, number of secondary xylem vessels remained unchanged (Fig. 2).

In the root of the radish plants with *RsCLE41* overexpression or treated by CLE41 the cells of the mechanical xylem parenchyma were absent. The same effect we observed in the radish roots with overexpression of *RsCLE2* or treated by CLE2 peptide. Conversely, *RsCLE19* overexpression or CLE19 peptide treatment increased the number of mechanical xylem parenchyma cells (Welch’s *t*-test *p*-value = 0.0092; *n* = 4). Therefore, A-type CLE peptides CLE2 and CLE19 presumably play different roles in the differentiation of secondary xylem elements, mainly of mechanical xylem parenchyma. Earlier, the negative effect of CLE41 on xylem differentiation was observed in *Arabidopsis* [7, 8]; Fiers et al. also revealed stimulatory effect of CLE19 on formation of extra xylem islands in *Arabidopsis* [32]; but the effect of CLE2 on xylem differentiation was not previously observed.

Secondly, radish roots with overexpression of *RsCLE41* or treatment by CLE41 peptide had increased number of meristematic foci in a central part of secondary xylem. According to literature data [26, 27], during the formation of storage root in radish some xylem parenchyma cells proliferate giving rise to meristematic foci—small areas of secondary cambium capable to divide and differentiate into phloem and xylem cells. In the roots with overexpression of *RsCLE41* or treated with CLE41 peptide these “meristematic foci” are enlarged and include small thin-walled cells, similar to cells of cambium, young vessels surrounding cells thick-walled xylem parenchyma cells and also phloem cells (Fig. 2). Therefore, in the storage root of radish, CLE41 can stimulate the proliferation of not only regular cambium, but also of cambium cells in the meristematic foci.

We also treated *R. raphanistrum* roots with exogenous CLE-peptides. In contrast to *R. sativus*, the effect of CLE-peptides on *R. raphanistrum* root structure was less pronounced, however, the general trend of the changes was the same. CLE19p slightly increased the number of mechanical xylem parenchyma, but there was no statistically significant difference in the number of xylem elements between control and CLE19-treated plants. CLE41 increased cambium cells number, however, no extra cambium foci were observed in the secondary xylem of CLE41-treated as well as in control *R. raphanistrum* plants. Treatment with CLE2p did not cause any changes in the number of xylem elements in *R. raphanistrum* roots. We speculate that different effects of CLE peptides on root structure of *R. sativus* that forms storage roots and *R. raphanistrum* may result from the differences in regulation of secondary root development in these radish species. Moreover, these two species might have initially different levels of other plant hormones interacting with CLE-peptides, which also could lead to different responses to exogenous CLE-peptide treatment.

### Influence of exogenous cytokinin and auxin on expression of *RsCLE* genes

It is well known that auxins and cytokinins are two main groups of phytohormones regulating the development of vascular system and secondary thickening of root. In the primary root of *Arabidopsis* these hormones demonstrate complementary patterns of distribution: auxins are concentrated in the differentiated xylem, while cytokinins are present in the cambium and phloem. Complementary auxin and cytokinin distribution is believed to be required for the proper development of the vascular system and to result from of cytokinin-dependent control of polar auxin transport and auxin-dependent repression of cytokinin signaling [36]. Cytokinins are necessary for induction of cambium in procambium and also cambium cell proliferation [37], auxin is also needed for proliferation of cambium—probably due to auxin-dependent control of *WOX4* gene expression [38]. Auxin transport and signaling components also play a key role in vascular cell specification [39].

So, there are two groups of regulators that control the development of vascular system and root secondary thickening—auxins and cytokinins along with CLE-peptides. It is probable that they can have some common targets—e.g. *WOX4* gene, and thereby, they may interact.

Previously, some data on the interaction between cytokinins and CLE-peptides or auxins and CLE peptides have been reported. In *M. truncatula*, it was discovered that synthetic cytokinin BAP has a stimulating effect on the expression of *MtCLE12* and *MtCLE13* genes which are central regulators of symbiotic nodule formation [12]. Conversely, *Arabidopsis* A-type CLE peptide AtCLE10 can stimulate cytokinin signaling by negative regulation of A-type ARR gene expression [15]. In rice, A-type *CLE* gene *OsCLE48* expression was induced by exogenous application of IAA [40]. In *Arabidopsis* lateral root formation some *AtCLEs* were up- or down-regulated by different hormones—auxin, ABA, brassinosteroids, salicylic acid and jasmonic acid, as well as by nutrients and stress [35]. However, almost nothing is known about the mechanism of interaction of CLE-peptides and other hormones.

**Fig. 4:**
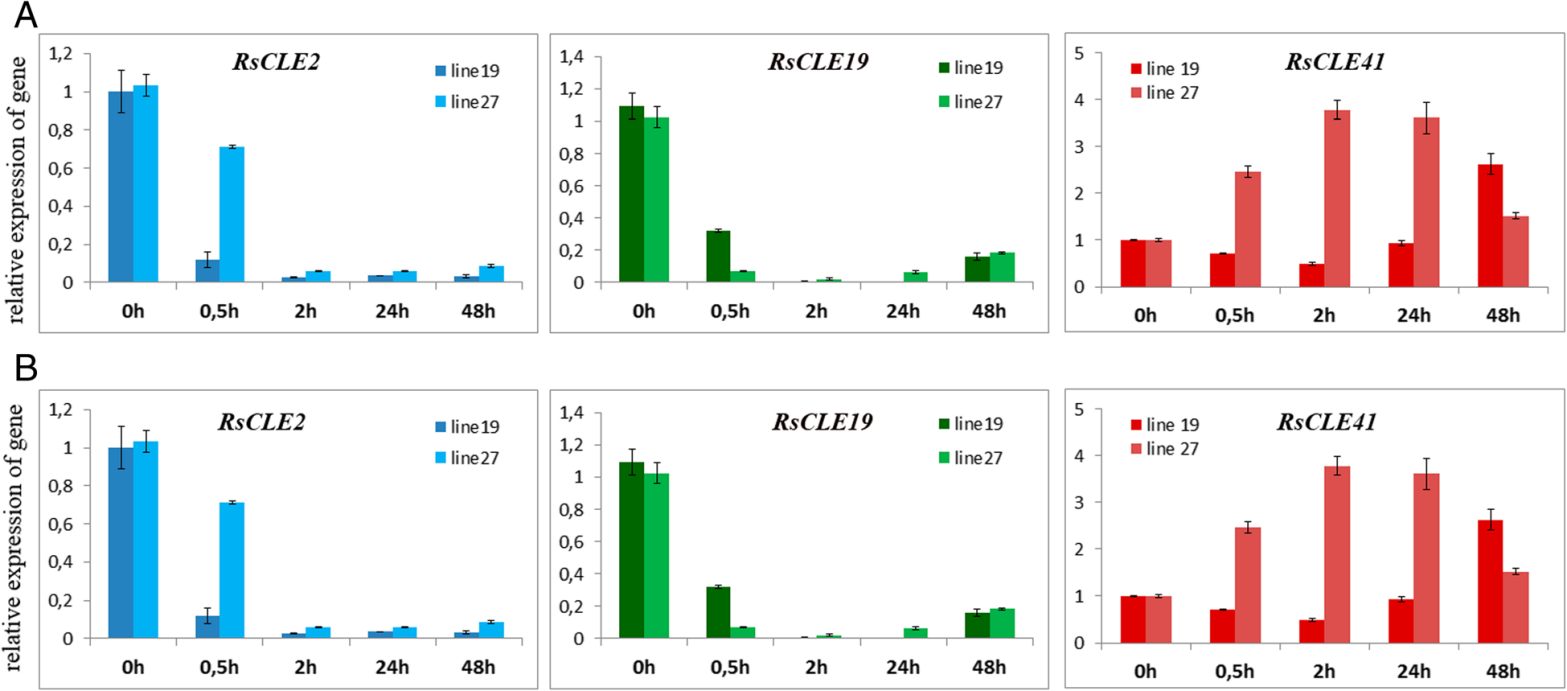
Effect of exogenous cytokinin (BAP, 10 mM) (**a**) and auxin (NAA, 10 mM) (**b**) on expression of *RsCLE2*, *19* and *41* genes in upper part of root of *Raphanus sativus* seedlings. Error bars indicate standard deviation of three technical repeats. (*P* < 0.05 - *, *P* < 0.01 - **, *P* < 0.001 - ***)

We measured the effect of exogenous auxin (NAA) and cytokinin (BAP) treatment on the expression of *RsCLEs* in upper part of the root and lower part of hypocotyl of *R. sativus* seedlings. We observed different expression dynamics for analyzed A-type (*RsCLE2*, *5*, *19*) and B-type (*RsCLE41*, *42*) *RsCLEs* in response to cytokinin treatment. Expression of A-type *RsCLEs* strongly decreased (ten times and more) after BAP treatment, even as early as 0.5 h after the treatment, whereas the expression of group B *RsCLEs* in response to BAP was not significantly altered (Fig. 4a). We also observed different expression dynamics for the same A- and B-type *RsCLEs* in response to treatment by auxin: expression levels of A-type *RsCLEs* increased (tenfold and more for *RsCLE19*, less than tenfold for *RsCLE2* and *RsCLE5*), while expression of B-type genes (*RsCLE41* and *42*) decreased dramatically (Fig. 4b).

Therefore, the same A- and B-type *RsCLEs* analyzed demonstrated quite different dynamics of expression in response to auxin and cytokinin: cytokinin seems to repress the expression of A-type *RsCLEs* but it did not influence the B-type *RsCLEs*; conversely, auxin seems to stimulate expression of A-type gene *RsCLE19* and negatively regulate both B-type *RsCLEs*.

Our results coincide with data on the role of cytokinin and B-type CLEs, and also auxin and A-type CLEs in the development of vascular system. It is known that auxin [41] and A-type CLEs such as AtCLE19 [32] stimulate xylem differentiation, whereas cytokinin [37] and B-type CLEs [11, 30] are necessary for cambium development. Taking that into account, we can suppose that these three groups of phytohormones can interact during the formation of plant vascular system and the development of radish storage root.

## Conclusions

Mechanisms underlying the formation of the storage root are poorly understood. In our study, we investigated the role of CLE peptides in the development of storage root of radish. Our data indicate that some CLE peptides such as CLE19, 41 and 2 may play a role in this process. Thus, the existing ideas about the functions of these peptides in plant development can be extended. Our findings on the effect of cytokinin and auxin on expression of genes encoding A- and B-types CLE peptides allow to suppose the interaction between different groups of phytohormones in the development of storage root of radish.

## Methods

### Plant material

*Raphanus raphanistrum* and two related inbred lines from a genetic collection of *Raphanus sativus* [21] 19 and 27, were used in this study.

### Plant growing conditions

For qRT-PCR and microscopy studies *R. sativus* and *R. raphanistrum* plants were grown in the ground. For transformation by *Agrobacterium rhizogenes* 7-day old *R. sativus* seedlings grown under aseptic culture conditions were used. For treatment by exogenous CLE peptides plants in hydroponic conditions were used. All plants were grown at 21 °C and 16 h photoperiod.

### Isolation of *RsCLE* genes

Total DNA of *R. sativus* was isolated from radish seedlings using the cetyltrimethylammonium bromide (CTAB) method [42]. To amplify the fragments of radish *CLE* genes, PCR was performed with primers designed for the conserve regions of corresponding genes of *Arabidopsis thaliana* and *Brassica rapa* using a VectorNTI Advance_10 (Invitrogen, United States) program (Additional file 4: Table S2). PCR-products were separated by electrophoresis in 1 % agarose gel containing ethidium bromide (0.1 %). Target fragments were isolated from the gel using a Cleanup Mini Kit (Evrogen, Russia) according to the manufacturer’s instructions and cloned into the pAL-TA vector (Evrogen, Russia). Transformation of chemically competent cells of *Escherichia coli* strain DH5α was carried outaccording to the protocol described in [43]. Transformants were selected on solidified LB medium containing 100 mg/L ampicillin and X-Gal. Plasmid DNA of selected transformant clones was isolated by Plasmid Miniprep Kit (Evrogen, Russia) and sequenced in SPbU Research Park, Center of Molecular and Cell Technologies. Sequence aligning of *Arabidopsis* and radish genes was performed using the AlignX program of VectorNTI Advance_10 (Invitrogen) software.

### qRT-PCR analysis

Total RNA was extracted by Purezol reagent (Bio-Rad, USA), purified with chloroform, and precipitated with isopropanol. The RNA pellet was washed three times with 80 % ethanol, dried under air flow in a laminar box, dissolved in sterile deionized water. RNA was treated with DNase (Syntol, Russia), subsequently purified with chloroform, reprecipitated with 0.3 M sodium acetate in the presence of ethanol and dissolved in sterile deionized water. RNA concentration was measured using the NanoDrop 2000c UV spectrophotometer (Thermo Scientific, USA) at 260 nm. For reverse transcription, 0.5 μg of RNA were used in all samples. RNA reverse transcription was performed using “Synthesis of cDNA first chain kit, version with oligo-dT” (Silex-M, Russia) according to the enclosed protocol.

The qRT-PCR experiments were done on a CFX-96 real-time PCR detection system with C1000 thermal cycler (Bio-Rad, USA), and Eva Green intercalating dye was used for detection (Syntol, Russia). Primers for qRT-PCR were designed to amplify 150–220 bp fragments. All reactions were performed in triplicate and averaged. Cycle threshold values were obtained with the accompanying software, and data were analyzed by 2^−ΔΔCt^ method [44]. Relative expression was normalized against constitutively expressed ubiquitin (*RsUBQ*) and glyceroaldehyde-3-phosphate dehydrogenase (*RsGAPDH*) genes [45]. Experiments were repeated three times with independent biological samples.

### Construction of vectors

PCR-fragments amplified on *R. sativus* DNA with primers for full-length CDS of *RsCLER2*, *19* and *41* were cloned to pENTR/D-TOPO vector (Invitrogen, USA) and after that transferred to pB7WG2D vector (Ghent, Belgium) for overexpression using the LR Clonase enzyme (Invitrogen, USA). This vector contains *GFP* gene under constitutive promoter for transgenic organs selection.

Constructs were introduced into *Agrobacterium rhizogenes* MSU440 strain via electroporation using Eppendorf Eporator® (Eppendorf, Germany).

### *Agrobacterium rhizogenes*-mediated plant transformation

*A. rhizogenes*-mediated plant transformation was performed as described previously [22]. To obtain sterile *R. sativus* seedlings, seeds were sterilized for 7 min with a mixture of 30 % hydrogen peroxide and 95 % ethanol (1:1), then washed with sterile distilled water and placed for germination on the Murashige-Skoog medium [46]. GFP-positive transgenic roots were selected using epifluorescent stereomicroscope Leica M205FA (Germany), all GFP-negative (nontransgenic) roots were cut off, and after that plants were placed into pots with vermiculite and cultivated at 21 °C with a 16-h photoperiod. After about 7 days, plants were transferred to pots with soil. After 30 days (rosette stage) roots were harvested and analyzed.

### Plant treatment with synthetic CLE peptides

For treatment with synthetic CLE peptides, *R. sativus* plants were grown in hydroponic system as described in [47]. CLE2p, CLE19p and CLE41p were used to treat plants, and CLE41def peptide with substitution of conserved G6 residue and non-hydroxylated prolin residues was used as a control (Additional file 5: Table S3). Synthetic peptides CLE2p, CLE19p, CLE41p and CLE41def of 95 % purities were obtained from ATG Service Gene (Russia). All peptides used were diluted to 10 mM stock solutions and stored at -20 °C. For plant treatment, stock solutions were added to hydroponic medium to working concentration 10 μM, and plant were cultivated in the medium with CLE peptide for 7 days.

### Seedlings treatment with auxin and cytokinin

For treatment with auxin or cytokinin, sterile *R. sativus* 7-day old seedlings were planted on solid MS medium supplied with 10 mM of synthetic auxin 1-Naphtaleneacetic acid (NAA) or synthetic cytokinin 6-Benzylaminopurine (BAP), and grown for 0.5, 2, 24 and 48 h. Seedlings planted on MS medium without hormones were used as a control.

### Histological analysis

Roots of soil-growing *R. sativus* and *R. raphanistrum* plants at 7^th^, 15^th,^ and 30^th^ days were fixed with 3 % paraformaldehyde, 0.25 % glutaraldehyde, 0.1 % Tween-20, 0.1 % Triton X-100 in 1/3 MTSB by vacuum infiltration for 10 min with subsequent incubation overnight at 4 °C. Samples were ethanol-dehydrated and embedded in agarose (3 %), and 50 μm sections were prepared with a Leica Vibratome VT-1200S (Leica, Germany). Samples were stained with 0.05 % wt/vol toluidine blue for 5 s and analyzed under Leica DM4000 microscope (Leica, Germany).

### Statistical methods

Welch’s unpaired *t*-test as implemented in R statistical environment (v. 3.0.2) was used to compare numbers of xylem elements. Gene expression across samples was compared using Welch’s *t*-test as implemented is R (3.0.2). Normality of distributions of data was tested using Shapiro-Wilk test in R (3.0.2).

## Additional files

Additional file 1: Table S1. Expression of *RsCLE* genes in the organs of *Raphanus raphanistrum* and *Raphanus sativus* 15-day old plants. (PDF 105 kb) Additional file 2: Figure S1. Expression of *RsCLE* genes in different organs of seedling in *Rapahnus sativus*. Expression levels are shown relative to the expression of *RsCLE1* found in the apex of 7-day old seedlings. Error bars indicate standard deviation of three technical repeats. (PDF 188 kb) Additional file 3: Figure S2. Expression of *RsCLE* genes in hypocotyls of *Rapahnus sativus* and *Raphanus sativus* (line 27) at different stage of development. Expression levels are shown relative to the expression found in the hypocotyl of 7-day old seedlings. Error bars indicate standard deviation of three technical repeats. (PDF 186 kb) Additional file 4: Table S2. Primers for identification of *RsCLE* genes. (PDF 179 kb) Additional file 5: Table S3. Synthetic CLE peptides used for treatment of radish plants. (PDF 287 kb)
