# Supplementary material for: Identification, expression, and functional analysis of CLE genes in radish (Raphanus sativus L.) storage root
Source: BMC Plant Biol. 2016 Jan 27;16(Suppl 1):7. doi: 10.1186/s12870-015-0687-y (PMC4895270; doi:10.1186/s12870-015-0687-y)
Supplement: Additional file 1: Table S1. — Expression of RsCLE genes in the organs of Raphanus raphanistrum and Raphanus sativus 15-day old plants. (PDF 105 kb) [file 12870_2015_687_MOESM1_ESM.pdf]

Table 1. Expression of *RsCLE* genes in the organs of *Raphanus raphanistrum* and *Raphanus sativus* 15-day old plants.

| Gene | Organ | Expression in radish | | Gene | Organ | Expression in radish | |
|---|---|---|---|---|---|---|---|
| | | *R. raphanistru* | *R. sativus* (line 27) | | | *R. raphanistrum* | *R. sativus* (line 27) |
| *RsCLE1* | Root | + | +/- | *RsCLE19* | Root | - | ++ |
| | Hypocotyl | +/- | - | | Hypocotyl | + | +/- |
| | Leaf | - | - | | Leaf | - | - |
| | Shoot apex | - | - | | Shoot apex | + | - |
| *RsCLE2* | Root | ++ | ++ | *RsCLE20* | Root | + | + |
| | Hypocotyl | + | +/- | | Hypocotyl | +/- | + |
| | Leaf | - | - | | Leaf | + | +/- |
| | Shoot apex | - | - | | Shoot apex | + | ++ |
| *RsCLE4* | Root | +/- | +/- | *RsCLE22* | Root | +/- | +/- |
| | Hypocotyl | - | - | | Hypocotyl | +/- | ++ |
| | Leaf | - | - | | Leaf | - | +/- |
| | Shoot apex | - | - | | Shoot apex | +/- | ++ |
| *RsCLE5* | Root | + | + | *RsCLE25* | Root | + | ++ |
| | Hypocotyl | ++ | ++ | | Hypocotyl | + | ++ |
| | Leaf | - | - | | Leaf | +/- | +/- |
| | Shoot apex | + | +/- | | Shoot apex | + | ++ |
| *RsCLE11* | Root | + | + | *RsCLE26* | Root | + | + |
| | Hypocotyl | + | + | | Hypocotyl | + | +/- |
| | Leaf | - | - | | Leaf | + | - |
| | Shoot apex | - | - | | Shoot apex | + | +/- |
| *RsCLE12* | Root | +/- | - | *RsCLE27* | Root | +/- | +/- |
| | Hypocotyl | - | - | | Hypocotyl | - | +/- |
| | Leaf | - | +/- | | Leaf | - | - |
| | Shoot apex | +/- | - | | Shoot apex | - | ++ |
| *RsCLE13* | Root | +/- | +/- | *RsCLE40* | Root | + | + |
| | Hypocotyl | - | - | | Hypocotyl | - | - |
| | Leaf | - | - | | Leaf | - | - |
| | Shoot apex | - | - | | Shoot apex | - | - |
| *RsCLE16* | Root | ++ | ++ | *RsCLE41* | Root | + | + |
| | Hypocotyl | ++ | ++ | | Hypocotyl | ++ | ++ |
| | Leaf | ++ | ++ | | Leaf | + | + |
| | Shoot apex | ++ | ++ | | Shoot apex | ++ | ++ |
| *RsCLE17* | Root | - | - | *RsCLE42* | Root | + | + |
| | Hypocotyl | - | - | | Hypocotyl | + | +/- |
| | Leaf | - | - | | Leaf | +/- | +/- |
| | Shoot apex | - | - | | Shoot apex | + | +/- |
