# Supplementary material for: Identification, expression, and functional analysis of CLE genes in radish (Raphanus sativus L.) storage root
Source: BMC Plant Biol. 2016 Jan 27;16(Suppl 1):7. doi: 10.1186/s12870-015-0687-y (PMC4895270; doi:10.1186/s12870-015-0687-y)

Figure 1. Expression of *RsCLE* genes in different organs of seedling in *Rapahnus sativus*.

Expression levels are shown relative to the expression of *RsCLE1* found in the apex of 7-day old seedlings.

Error bars indicate standard deviation of three technical repeats.

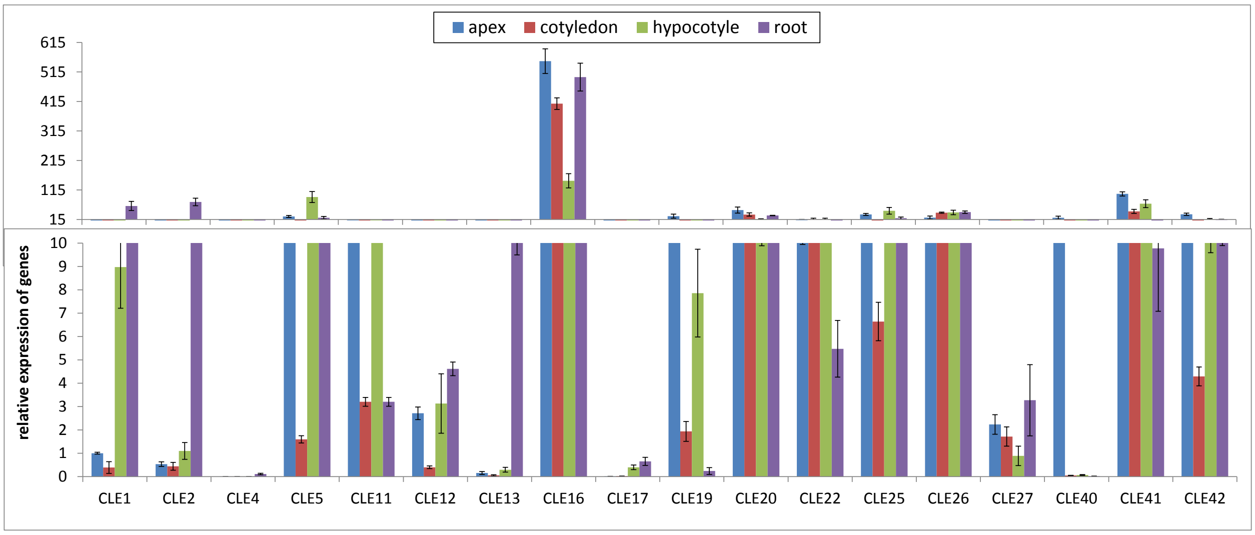

Supplement: Additional file 2: Figure S1. — Expression of RsCLE genes in different organs of seedling in Rapahnus sativus. Expression levels are shown relative to the expression of RsCLE1 found in the apex of 7-day old seedlings. Error bars indicate standard deviation of three technical repeats. (PDF 188 kb) [file 12870_2015_687_MOESM2_ESM.pdf]
