# Supplementary material for: Identification, expression, and functional analysis of CLE genes in radish (Raphanus sativus L.) storage root
Source: BMC Plant Biol. 2016 Jan 27;16(Suppl 1):7. doi: 10.1186/s12870-015-0687-y (PMC4895270; doi:10.1186/s12870-015-0687-y)

Figure 2. Expression of *RsCLE* genes in hypocotyls of *Rapahnus sativus* and *Raphanus sativus* (line 27) at different stage of development. Expression levels are shown relative to the expression found in the hypocotyl of 7-day old seedlings.

Error bars indicate standard deviation of three technical repeats.

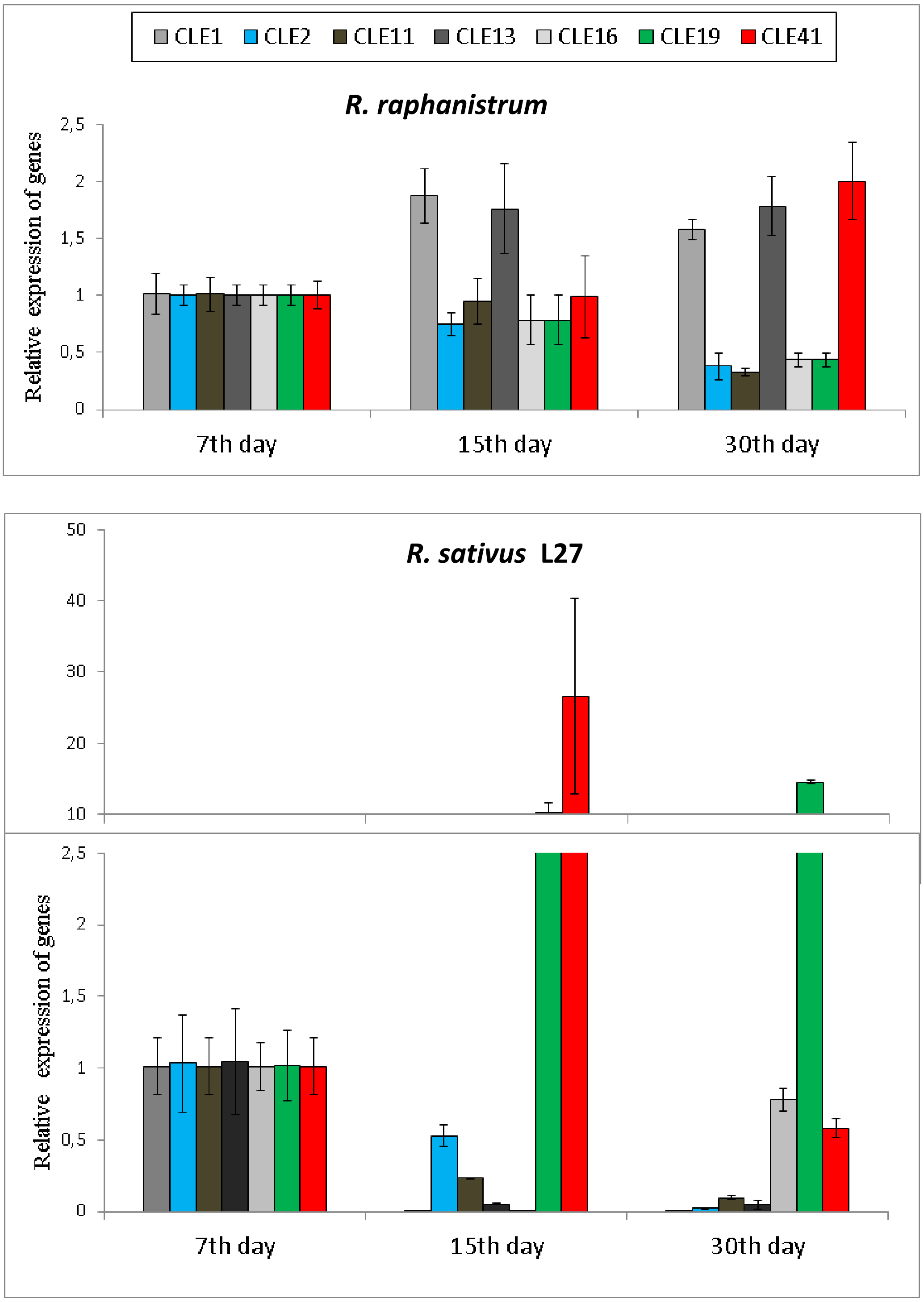

Supplement: Additional file 3: Figure S2. — Expression of RsCLE genes in hypocotyls of Rapahnus sativus and Raphanus sativus (line 27) at different stage of development. Expression levels are shown relative to the expression found in the hypocotyl of 7-day old seedlings. Error bars indicate standard deviation of three technical repeats. (PDF 186 kb) [file 12870_2015_687_MOESM3_ESM.pdf]
