# Supplementary material for: Identification, expression, and functional analysis of CLE genes in radish (Raphanus sativus L.) storage root
Source: BMC Plant Biol. 2016 Jan 27;16(Suppl 1):7. doi: 10.1186/s12870-015-0687-y (PMC4895270; doi:10.1186/s12870-015-0687-y)
Supplement: Additional file 4: Table S2. — Primers for identification of RsCLE genes. (PDF 179 kb) [file 12870_2015_687_MOESM4_ESM.pdf]

Table 2. Primers for identification of *RsCLE* genes.

Gene	Primers	Gene	Primers
RsCLE1	F atggctaacatgaaagtttggctg<br>R tcagtgatgacgagggtcggga	*RsCLE19*	F attggcttcttctctcctgattcttgc<br>R ttacctgttgtggagtggatttgga
RsCLE2	F atggctaagttaagcttcactttctg<br>R ctggtgatgttgtgggtcgggtc	*RsCLE20*	F gtcgtcctcgtcttctctgtctcat<br>R tcatcgcttgtttgtaaagga
RsCLE4	F atggcaagtttcaacaagttatggg<br>R tttagtgatgtctagggtcc	*RsCLE22*	F atgagaaataactactccagaagaaaacc<br>R ttatcaactgtgcgaaggattagg
RsCLE5	F tctctctctcaactcccacccctc<br>R gaaaccctgtcggaactcactaaaa	*RsCLE25*	F atgggtggaagtggcattagag<br>R tcatactcgtgttggtcgtctcg
RsCLE11	F tgcgtatgtcaccccaccca<br>R accacccgttaatcatcattgt	*RsCLE26*	F acccagaagacaagacagaaagaaga<br>R ctagttatggagaggatctggacca
RsCLE12	F atgcttagaatttcttcatcatcttcc<br>R ccaatcccgttgtaccattga	*RsCLE27*	F atggcggcgatgaaatacaaagg<br>R ctatggagtaaaaggaatgtgtttatgatgaa
RsCLE13	F atagccacgagtagagtctcccac<br>R tcagtcgtgtaagcggtttg	*RsCLE40*	F atggcaacatcaaatgaccaaacca<br>R ctgaagattcgaggagaaccctcttg
RsCLE16	F acaaaagaagaagaagaagacgagcag<br>R agttgtggagaggatttggacctg	*RsCLE41*	F tatttggtataacatgtgatgagatc<br>R ctattggagatgggatttggac
RsCLE17	F atgactcaagtgttggtacgaaga<br>R ttagttgtggagaggattgggac	*RsCLE42*	F atgggtggaagtggcattagag<br>R tcatactcgtgttggtcgtctcg
