# Supplementary material for: Identification, expression, and functional analysis of CLE genes in radish (Raphanus sativus L.) storage root
Source: BMC Plant Biol. 2016 Jan 27;16(Suppl 1):7. doi: 10.1186/s12870-015-0687-y (PMC4895270; doi:10.1186/s12870-015-0687-y)
Supplement: Additional file 5: Table S3. — Synthetic CLE peptides used for treatment of radish plants. (PDF 287 kb) [file 12870_2015_687_MOESM5_ESM.pdf]

Table 3. Synthetic CLE peptides used for treatment of radish plants.

Peptide	Sequence*
CLE2	RLShPGGhPDPQHH
CLE19	RVIhPTGhPNPLHN
CLE41	HEVhPSGhPNPISN
CLE41-def (control)	HEVPSGPNAISN

*hP - hydroxyproline
